# A predictive model for Epstein-Barr virus-associated hemophagocytic lymphohistiocytosis

**DOI:** 10.3389/fimmu.2024.1503118

**Published:** 2024-12-05

**Authors:** Rui Huang, Dan Wu, Ling Wang, Ping Liu, Xiaoru Zhu, Leqiu Huang, Mengmeng Chen, Xin Lv

**Affiliations:** ^1^ Clinical Laboratory, Children’s Hospital Affiliated to Shandong University, Jinan, Shandong, China; ^2^ Clinical Laboratory, Jinan Children’s Hospital, Jinan, Shandong, China

**Keywords:** Epstein-Barr virus infections, lymphohistiocytosis, hemophagocytic, infectious mononucleosis, pediatrics, nomograms

## Abstract

**Background:**

Epstein-Barr virus-associated hemophagocytic lymphohistiocytosis (EBV-HLH) is a severe hyperinflammatory disorder induced by overactivation of macrophages and T cells. This study aims to identify the risk factors for the progression from infectious mononucleosis (EBV-IM) to EBV-HLH, by analyzing the laboratory parameters of patients with EBV-IM and EBV-HLH and constructing a clinical prediction model. The outcome of this study carries important clinical value for early diagnosis and treatment of EBV-HLH.

**Methods:**

A retrospective analysis was conducted on 60 patients diagnosed with EBV-HLH and 221 patients diagnosed with EBV-IM at our hospital between November 2018 and January 2024. Participants were randomly assigned to derivation and internal validation cohorts in a 7:3 ratio. LASSO regression and logistic regression analyses were employed to identify risk factors and construct the nomogram.

**Results:**

Ferritin (OR, 213.139; 95% CI, 8.604-5279.703; P=0.001), CD3^-^CD16^+^CD56^+^% (OR, 0.011; 95% CI, 0-0.467; P=0.011), anti-EBV-NA-IgG (OR, 57.370; 95%CI, 2.976-1106.049; P=0.007), IL-6 (OR, 71.505; 95%CI, 2.118-2414.288; P=0.017), IL-10 (OR, 213.139; 95% CI, 8.604-5279.703; P=0.001) were identified as independent predictors of EBV-HLH. The prediction model demonstrated excellent discriminatory capability evidenced by an AUC of 0.997 (95% CI,0.993-1.000). When visualized using a nomogram, the ROC curves for the derivation and validation cohorts exhibited AUCs of 0.997 and 0.993, respectively. These results suggested that the model was highly stable and accurate. Furthermore, calibration curves and clinical decision curves indicated that the model possessed good calibration and offered significant clinical benefits.

**Conclusions:**

The nomogram, which was based on these five predictors, exhibited robust predictive value and stability, thereby can be used to aid clinicians in the early detection of EBV-HLH.

## Introduction

The Epstein-Barr virus (EBV), classified within the subfamily γ of the Herpesviridae family, is a double-stranded DNA virus. Transmission of EBV primarily occurs through saliva, although it can also be transmitted via blood transfusion, sexual intercourse, and allografts. Initial infections in individuals under the age of 6 often present as asymptomatic or with mild upper respiratory symptoms, while approximately 50% of adolescents develop infectious mononucleosis (IM) (1). IM is a benign, self-limiting disease resulting from EBV infection. Common clinical presentations include fever, tonsillitis, and enlarged cervical lymph nodes, often accompanied by hepatosplenomegaly and peripheral blood heterogeneous lymphocytosis (2). The majority of cases have a favorable prognosis, although a minority of patients may develop multi-systemic complications, such as EBV-associated hemophagocytic syndrome(HPS) (3).

HPS, also known as hemophagocytic lymphohistiocytosis (HLH), is a severe condition characterized by immune system overactivation, leading progressive deterioration with immune dysfunction in multiple organs. HLH is distinguished by the excessive activation of macrophages and T cells, which results in the release of high levels of inflammatory cytokines (4). Clinical presentations of HLH include persistent fever, hepatosplenomegaly, pancytopenia, and phagocytosis within the bone marrow (1, 5).HLH is classified as either “primary” or “secondary” based on the underlying factor. Primary HLH is an autosomal or sex chromosome recessive disorder resulting from potential gene mutations, with 12 causative genes currently identified in association with primary HLH (6, 7). Based on the specific gene defects, primary HLH is classified as familial HLH (FHL) and immune-deficiency syndrome-associated HLH. Conversely, Secondary HLH is mainly linked to infection, malignancy, and autoimmunity, which are referred to as infection-associated hemophagocytic syndrome (IAHS), malignancy-associated hemophagocytic syndrome (MAHS), and macrophage activation syndrome (MAS), respectively (8). Infection-related HLH represents the most prevalent form of secondary HLH, with infectious agents such as bacteria, fungi, viruses, and protozoa implicated. Herpesvirus infections, specifically EBV infection-associated HLH (EBV-HLH), are a prominent form of secondary HLH. They account for approximately 70% of infection-associated HLH cases, with a higher prevalence among Asian children and adolescents (9).

In clinical practice, the initial symptoms of EBV-HLH patients are often atypical and challenging to identify, with some patients presenting with symptoms resembling infectious mononucleosis. However, the disease progresses rapidly, leading to high mortality rates among patients who delay seeking medical treatment or receive an unclear diagnosis. Therefore, it is imperative to compile and analyze the clinical readouts to improve the identification and diagnosis of EBV-HLH (10, 11).

In this retrospective study, an analysis of laboratory data was conducted to identify potential predictors of EBV-HLH in pediatric patients diagnosed with EBV-HLH or EBV-IM (12). Recognizing patients at risk of developing EBV-HLH is crucial for timely intervention. Therefore, examining the clinical risk factors associated with the development of EBV-HLH in patients with EBV infection is essential for early diagnosis and treatment.

## Materials and methods

### Patients

This retrospective study examined pediatric patients of EBV-IM and EBV-HLH at the Children’s Hospital Affiliated to Shandong University from November 2018 to January 2024. Clinical data was collected, including gender, age, serum cytokines (IL-2, IL-4, IL-6, IL-10, IFN-γ, and TNF-α), lymphocyte subsets, EBV DNA copy number, ferritin (FRT), lactate dehydrogenase (LDH), hydroxybutyrate dehydrogenase (HBDH) and EBV specific antibodies (anti-EBV-VCA-IgM, anti-EBV-VCA-IgG, anti-EBV-EA-IgG, and anti-EBV-NA-IgG). All patients’ blood samples were obtained for laboratory testing upon their first visits to the hospital. The EBV-HLH disease development happened in a time frame of 5-14 days. The study protocol was approved by our hospital’s institutional review boards (IRB number: SDFE-IRB/T-2024061) and followed the guidelines outlined in the Declaration of Helsinki.

### Diagnostic criteria

Patients meeting the HLH-2004 criteria and displaying active EBV infection were classified as having EBV-HLH (13). Diagnosis of HLH required the meeting at least five of the eight criteria: (1) fever; (2) splenomegaly; (3) cytopenia affecting two or more blood cell lineages (hemoglobin<90g/L, platelets<100×10^9^/L, and/or neutrophils <1.0×10^9^/L); (4) hypertriglyceridemia(≥265mg/dL) and/or hypofibrinogenemia (≤150g/dL); (5) hematophagy has been discovered in the bone marrow, spleen, liver, and lymph nodes; (6) natural killer (NK) cell activity is low or undetectable; (7) ferritin levels≥500μg/L; (8) increased interleukin-2 receptor levels (soluble CD25). Patients with primary HLH were excluded from the study, as it encompasses familial HLH and HLH caused by genetic variants of RAB27A, LYST, AP3B1, SH2D1A, and BIRC4.

Diagnosis of EBV-IM involves a combination of clinical symptoms and laboratory results (14), with key clinical indicators being: (1) fever; (2) pharyngeal tonsillitis; (3) cervical lymph node enlargement; (4) splenomegaly; (5) hepatomegaly; (6) eyelid edema. Biomarkers of EBV-IM include: (1) positivity for anti-EBV-VCA-IgM and anti-EBV-VCA-IgG antibodies and negativity for anti-EBV-NA-IgG anti-bodies; (2) negativity for anti-EBV-VCA-IgM antibodies but positivity for anti-EBV-VCA-IgG and low-affinity antibodies; (3) anti-EBV-VCA-IgG levels increased≥4-fold in two serum samples; (4) positivity for EBV DNA by polymerase chain reaction. A combination of three clinical indicators and one nonspecific laboratory test must be met for the diagnosis of EBV-IM.

### Measurement of cytokines, lymphocyte subsets, ferritin, LDH, HBDH and EBV-specific antibodies

Cytokine levels were assessed using the Cytokine Combination Assay Kit II (CEGER, China) (15).Lymphocyte subpopulation levels were determined with the Lymphocyte Subpopulation Assay Kit (BD, USA). Lactate dehydrogenase levels were measured through Lactate Dehydrogenase Assay Kit (Zhongya, China). HBDH levels were determined using the α-hydroxybutyrate dehydrogenase assay kit (Zhongya, China). EB-specific antibodies were measured by an EBV antibody detection kit (LIAISON, Italy). Ferritin was measured by a ferritin assay kit (SIEMENS, Germany). All measurement results were derived from the patients’ initial visits to our hospital.

### Prediction model development and validation

We performed a univariate analysis to analyze clinicopathological characteristics differences between the HLH and IM groups. Variables with statistically significant differences from the above analysis were used as candidate variables for least absolute shrinkage and selection operator (LASSO) regression and multivariate logistic regression to create a nomogram.

The appropriate cutoff values for variables were determined by receiver operating characteristic (ROC) analysis and the maximal Youden index. The area under the curve (AUC) was calculated to evaluate the nomogram’s discriminative ability. Meanwhile, the calibration curve and Hosmer–Lemeshow test were used to reduce the overfitting bias and assess the goodness of fit by comparing the actual probabilities and the probabilities predicted by our nomogram. To estimate the net benefits of the nomogram model under different threshold probabilities, decision curve analysis (DCA) was performed.

### Statistical method

SPSS 26.0 (SPSS Inc., Chicago, IL, USA) and R software (v4.0.1) were used for statistical analysis. The difference was statistically significant when the *P*-value was <0.05. The Wilcoxon rank sum test (continuous variables with skewed distributions) and the Pearson chi-square test (categorical variables) were used to determine statistical differences between the EBV-HLH and EBV-IM groups.

In order to filter the variables for prediction, LASSO regression was performed in the derivation cohort and the importance of these variables was compared by using the “glment package” and the “DALEX package”. The minimum error of lambda (l) values in LASSO regression were obtained as a criterion to screen the variables by ten‐fold cross‐validation. Multivariate logistic regression analysis was performed to estimate odds ratio (OR) with 95% confidence intervals (95% CI) and to identify independent predictor variables for EBV-HLH. The performance of this predictive model was evaluated by using ROC curves (“pROC” package), calibration curves (“RMS” package), and DCA (“rmda” package). A nomogram was constructed based on the five predictors.

## Results

### Patients’ characteristics

Out of the 281 children in the study, 60 were diagnosed with EBV-HLH and 221 with only EBV-IM. These patients were divided into derivation and internal validation cohorts using simple randomization at a ratio of 7:3, with 203 cases in the derivation cohort and 78 cases in the internal validation cohort. Patient characteristics can be found in **Supplementary Table S1**. The incidence of HLH was 20.2% in the derivation cohort and 24.36% in the validation cohort, with no significant difference in variables between the two cohorts (**Supplementary Table S1**).

### Independent predictors for EBV-HLH and model development

**Figure 1:**
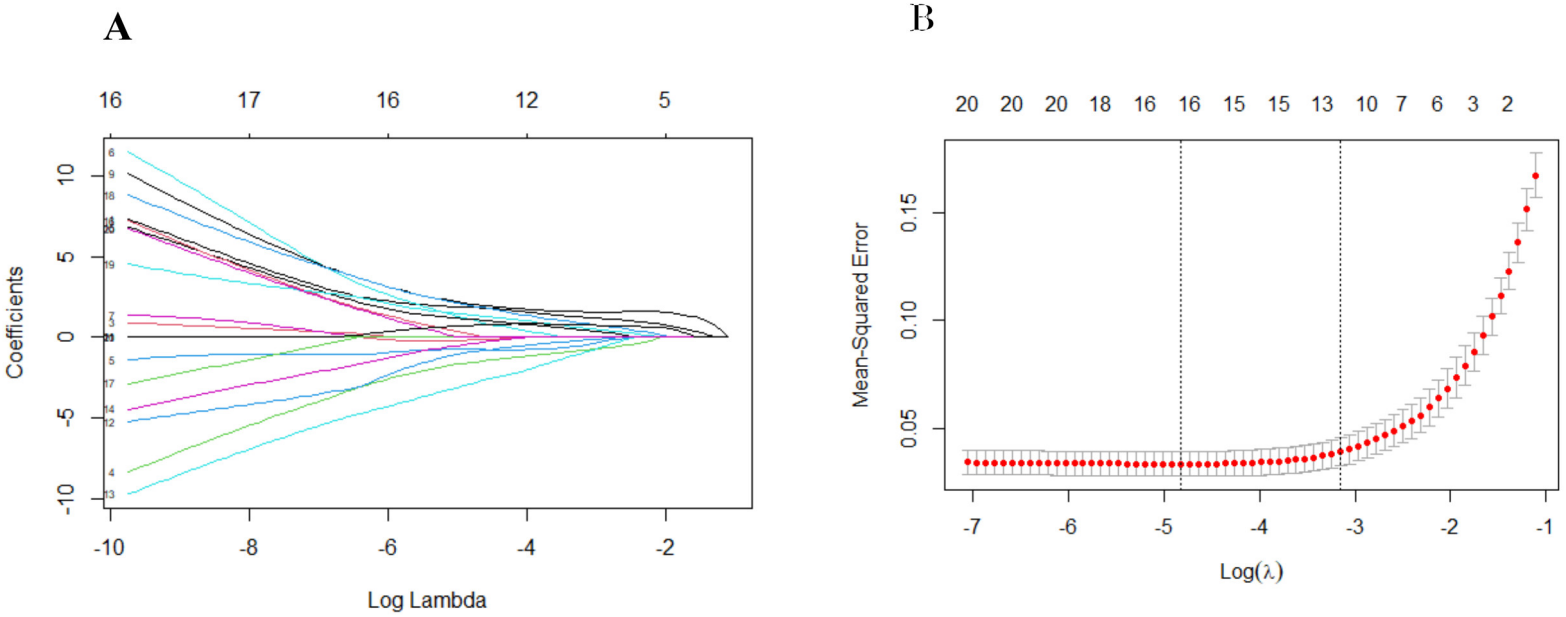
Screening of 21 variables based on Lasso regression. **(A)** The variation characteristics of the coefficient of variables; **(B)** the selection process of the optimum value of the parameter log(λ) in the Lasso regression model and 12 variables were selected for further logistic regression analysis.

We performed univariate regression analysis in the derivation cohort and identified predictors that were highly associated with EBV-HLH. All the variables showed statistically significant differences, with the exception of gender and IL-2 (**Table 1**). To build the optimal EBV-HLH risk prediction model, we conducted LASSO regression analysis of these variables, with twelve variables selected through cross-validation: ferritin, CD3^+^CD8^+^ T cell ratio (%), the ratio of CD4^+^ and CD8^+^T cells (CD4^+^/CD8^+^), CD3^-^CD16^+^CD56^+^ T cell ratio (%), HBDH, EBV-DNA copy number, anti-EBV-VCA-IgM, anti-EBV-VCA-IgG, anti-EBV-NA-IgG, IL-6, IL-10 and IFN-γ (**Figure 1**).

**Table 1:** Univariate analysis of the derivation cohort.

Variables		EBV-HLH(N=41)	EBV-IM(N=162)	*P*-value
Gender	Female	19	67	0.564
	Male	22	95	
Age	Age ≤ 2.125	19	16	0.000
	Age>2.125	22	146	
Ferritin	Ferritin ≤ 250	7	154	0.000
	Ferritin>250	34	8	
CD3^+^%	CD3^+^%≤91.705	35	155	0.016
	CD3^+^%>91.705	6	7	
CD3^+^CD4^+^%	CD3^+^CD4^+^%≤21.115	15	126	0.000
	CD3^+^CD4^+^%>21.115	26	36	
CD3^+^CD8^+^%	CD3^+^CD8^+^%≤54.975	26	36	0.000
	CD3^+^CD8^+^%>54.975	15	126	
CD4^+^/CD8^+^	CD4^+^/CD8^+^≤0.315	11	109	0.000
	CD4^+^/CD8^+^>0.315	30	53	
CD3^-^CD16^+^CD56^+^%	CD3^-^CD16^+^CD56^+^%≤6.295	35	34	0.000
	CD3^-^CD16^+^CD56^+^%>6.295	6	128	
CD3^-^CD19^+^%	CD3^-^CD19^+^%≤14.02	23	137	0.000
	CD3^-^CD19^+^%>14.02	18	25	
LDH	LDH ≤ 640	10	157	0.000
	LDH>640	31	5	
HBDH	HBDH ≤ 458.5	10	157	0.000
	HBDH>458.5	31	5	
EBV DNA copy No.	EB-DNA copy No.<10^3	10	140	0.000
	EB-DNA copy No.≥10^3	31	20	
anti-EBV-VCA-IgM	anti-EBV-VCA-IgM<20	12	0	0.000
	anti-EBV-VCA-IgM≥20	29	162	
anti-EBV-VCA-IgG	anti-EBV-VCA-IgG<20	8	154	0.000
	anti-EBV-VCA-IgG≥20	33	8	
anti-EBV-EA-IgG	anti-EBV-EA-IgG<10	18	144	0.000
	anti-EBV-EA-IgG≥10	23	18	
anti-EBV-NA-IgG	anti-EBV-NA-IgG <5	14	148	0.000
	anti-EBV-NA-IgG ≥5	27	14	
IL-2	IL-2<2.57	29	143	0.070
	IL-2≥2.57	12	29	
IL-4	IL-4<0.775	4	158	0.000
	IL-4≥0.775	37	4	
IL-6	IL-6<15.95	8	154	0.000
	IL-6≥15.95	33	8	
IL-10	IL-10<26.515	9	153	0.000
	IL-10≥26.515	32	9	
TNF-α	TNF-α<1.210	3	159	0.000
	TNF-α≥1.210	38	3	
IFN-γ	IFN-γ<12.87	8	154	0.000
	IFN-γ≥12.87	33	8	

**Figure 2:**
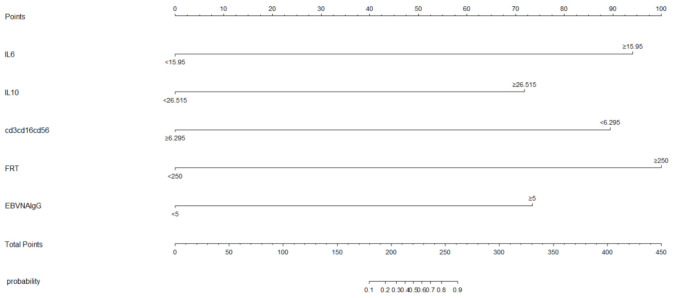
The nomogram for prediction of EBV-HLH.

**Figure 3:**
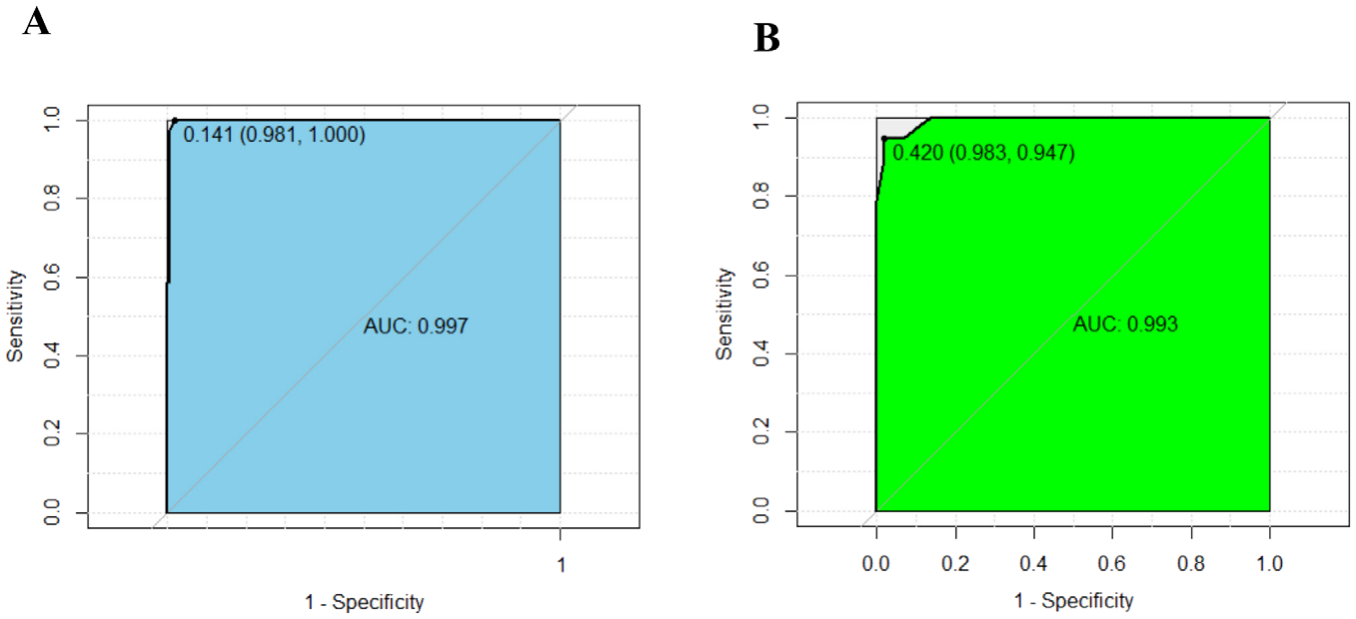
ROC curves show excellent discrimination ability for predicting EBV-HLH in the derivation **(A)** and internal validation cohorts **(B)**. The cutoff values were 0.141 (AUC:0.997, sensitivity: 0.981, specificity: 1.000), and 0.420 (AUC:0.993, sensitivity: 0.983, specificity: 0.947).

**Figure 4:**
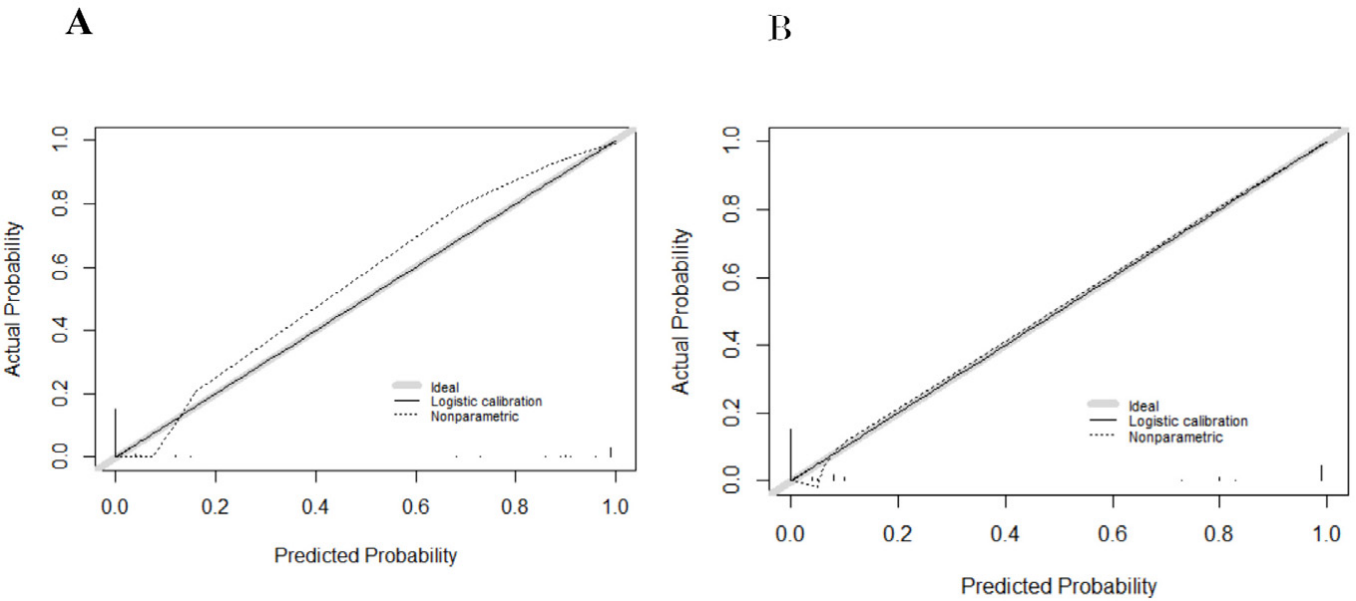
Calibration curves of the derivation cohort **(A)** and internal validation cohort **(B)**.

We subjected these variables to multivariate logistic regression analysis and identified five variables to be included in the predictive model: ferritin (OR, 213.139; 95% CI, 8.604-5279.703; *P*=0.001), CD3^-^CD16^+^CD56^+^% (OR, 0.011; 95% CI, 0-0.467; *P*=0.011), anti-EBV-NA-IgG (OR, 57.370; 95%CI, 2.976-1106.049; *P*=0.007), IL-6 (OR, 71.505; 95%CI, 2.118-2414.288; *P*=0.017), IL-10 (OR, 213.139; 95% CI, 8.604-5279.703; *P*=0.001) (**Table 2**). We have made dotplots to show the different distributions of these parameters between EBV-IM and EBV-HLH (**Supplementary Figure S1**). These predictors were represented in a nomogram (**Figure 2**), where each was assigned a point value between 0 and 100. By summing these points and locating them on the total point scale, the corresponding probability of EBV-HLH can be determined. The generated ROC curve for the derivation cohort demonstrated the predictive model’s excellent discriminatory ability, with an AUC of 0.997 (95% CI, 0.993-1.000) (**Figure 3A**). The calibration curves showed good agreement between predicted and actual EBV-HLH presence (**Figure 4A**), and the Hosmer-Lemeshow test showed no deviation from a good fit. The cutoff value to distinguish the presence of EBV-HLH in the derivation cohort is 0.141.

**Table 2:** Multivariate analysis of the derivation cohort.

Parameters	β	Wald χ 2	*P*-value	OR (95%CI)
Ferritin	5.362	10.721	0.001*	213.139(8.604-5279.703)
CD3^+^CD8^+^%	-0.029	0.000	0.996	0.972(0.000-31069.361)
CD4^+^/CD8^+^	2.559	3.098	0.078	12.928(0.748-223.458)
CD3^-^CD16^+^CD56^+^%	-4.479	5.576	0.018*	0.011(0-0.467)
HBDH	2.447	1.959	0.162	11.555(0.375-355.605)
EBV-DNA copy No.	0.931	0.256	0.613	2.537(0.069-93.236)
anti-EBV-VCA-IgM	-14.174	0.000	0.999	0.000(0.000-0.000)
anti-EBV-VCA-IgG	-26.101	0.000	0.998	0.000(0.000-0.000)
anti-EBV-NA-IgG	4.050	7.195	0.007*	57.370(2.976-1106.049)
IL-6	4.270	5.654	0.017*	71.505(2.118-2414.288)
IL-10	4.099	7.574	0.006*	60.296(3.254-1117.280)
IFN-γ	0.392	0.059	0.809	1.479(0.062-35.326)

*P<0.05.

### Internal validation and risk stratification

**Figure 5:**
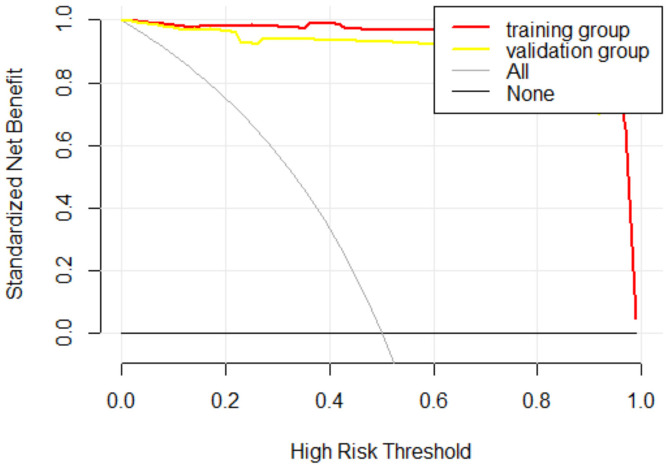
Decision curve of the derivation and validation cohorts.

The internal validation cohort was employed to evaluate the predictive accuracy of the model. **Figure 3B** illustrated the ROC curve, which demonstrated an AUC of 0.993 (95% CI, 0.980-1.000). The calibration curves indicated a high degree of concordance between the observed and predicted probabilities of EBV-HLH (**Figure 4B**). The Hosmer-Lemeshow test yielded a *P*-value of 0.985, indicating no significant difference. Decision curve analysis indicated that the nomogram was likely to yield substantial net benefits across a range of threshold probabilities for EBV-HLH (**Figure 5**).

Based on the constructed nomogram, a novel risk stratification model for EBV-HLH was constructed (**Table 3**). The risk point assigned to various predictors were as follows: ferritin>250ug/L (13 points), CD3^-^CD16^+^CD56^+^%>6.295 (-22 points), anti-EBV-NA-IgG≥5U/ml (10 points), IL-6≥15.95pg/ml (11 points) and IL-10≥26.515pg/ml (10 points). The total risk points for each patient were calculated by summing the individual risk points of these predictors. Based on the distribution characteristics of EBV-HLH prevalence, a cutoff value was established to stratify patients into three distinct subgroups: patients with total points of -22, -12, -11, -1, or 0 were assigned to the low-risk subgroup; patients with total points>11 were classified as the high-risk subgroup; and the remaining patients were defined as the moderate-risk subgroup. A significant difference was found among the three subgroups in terms of EBV-HLH prevalence.

**Table 3:** The risk stratification of patients and the incidence rate of EBV-HLH.

Total points	Risk stratification	EBV-HLH		The incidence rate of EBV-HLH (%)	*P*-value
		NO	YES		
-22, -12, -11, -1, 0	Low-risk	197	1	0.5	0.000
1, 9, 10, 11	Moderate-risk	20	1	4.8	
>11	High-risk	4	58	93.5	

## Discussion

Most primary EBV infections manifest as IM in pediatric populations, with a minority of these cases progressing to EBV-associated hemophagic syndrome (16). The clinical course of hemophagic syndrome is often rapid and severe, with a notably poor prognosis. However, early diagnosis and prompt therapeutic interventions can significantly improve survival outcomes (17).

It has been postulated that deficiencies in natural killer (NK) cell and cytotoxic T lymphocyte (CTL)-mediated cytotoxicity may precipitate an exaggerated or inadequate immune response, culminating in which could give rise to a cytokine storm and multisystem-involved inflammation inflammatory involvement (18). Impaired immune regulation can cause an excessive proliferation of immunoreactive cells, producing a surge of inflammatory cytokines that lead to If the patient’s immune regulation is compromised, resulting in an excessive proliferation of immunoreactive cells, these cells can produce a surge of inflammatory cytokines, culminating in HLH (19).

Lymphocyte subpopulation analysis serves as a crucial indicator for assessing both cellular and humoral immunity. This analysis can provide a snapshot of the current state of the immune system, aiding in the diagnosis of specific diseases and contributing to etiological analysis and prognostic evaluation (20). CD3^+^ T cells encompass the total population of T cells. CD4^+^ T cells, also known as helper T cells, play a crucial role in both cellular and humoral immunity. CD8^+^ T cells, which include CTLs and suppressor T cells, primarily function to eliminate target cells and secrete suppressor factors, thereby modulating the immune response. The CD4^+^/CD8^+^ ratio serves as a sensitive indicator for clinical diagnosis of human immune dysfunction (21–23).CD3^-^CD16^+^CD56^+^ cells refer to natural killer (NK) cells, which are vital components of the immune system. NK cells are not only involved in anti-tumor and anti-viral activities, but also implicated in hypersensitivity reactions and autoimmune diseases. NK cells are adept at recognizing target cells and mediating cytotoxic responses. CD3^-^CD19^+^ cells, also known as B lymphocytes, are responsible for antibody secretion, antigen presentation, and cytokine production. They play a critical role in regulating the immune system and inflammatory responses.

Our results indicate that CD3^-^CD16^+^CD56^+^ cells (NK cells) are important within this predictive model. NK cells constitute a major component of the non-specific immune system. The proportion of NK cell immunity in EBV-HLH is significantly lower compared to EBV-IM, indicating a reduced non-specific NK cell response in patients with EBV-HLH. NK cells eliminate virus-infected or malignant cells using a variety of mechanisms, some of which rely on cytotoxic granule-mediated killing. Granule-mediated cytotoxicity is carried out through the polarized delivery of cytotoxic granule contents to the immunologic synapse, extrusion of contents into the space shared with a target cell, and perforin-mediated entry into the target cell where cytotoxic granule contents induce target-cell apoptosis.

In patients with familial hemophagocytic lymphohistiocytosis (HLH) types 2 to 5, as well as those with pigmentary disorders associated with HLH, granule-mediated cytotoxicity is compromised. The prolonged duration of synapse formation between cytotoxic lymphocytes—deficient in perforin or granzymes—and target cells results in the excessive production of inflammatory cytokines. Furthermore, antigen-presenting cells accumulate and persistently stimulate T cells, thereby exacerbating T-cell activation and proliferation. This initiates a self-perpetuating cycle of lymphohistiocytic proliferation and hypercytokinemia, ultimately culminating in extensive tissue damage and hyperinflammatory syndrome of HLH (24). On the other hand, the percentage of CD4^+^ and CD8^+^ T cells, the CD4^+^/CD8^+^ cell ratio, and the percentage of CD3^-^CD19^+^ cells were not effective in distinguishing between the two disorders due to their lower sensitivity, supporting the previous findings. Further research is needed to elucidate the molecular mechanism underlying the immune responses in IM and HLH upon EBV infection.

The pathophysiological abnormalities in the immune mechanism of HLH are characterized by the dysregulated activation of T cells, particularly CD8^+^ cytotoxic T cells, and macrophages. This dysregulation results in the excessive production of cytokines, contributing to the clinical manifestations of the disease (25, 26). Raj et al. elucidated that EBV infection led to the augmented synthesis and secretion of cytokines, including IFN-γ, IL-10, and granulocyte-macrophage colony-stimulating factor (GM-CSF). These elevated cytokine levels further stimulated the activation of cytotoxic T-cell (CTL) and macrophages (27–30). The activated macrophages, in turn, secreted substantial quantities of pro-inflammatory cytokines such as IFN-γ and TNF-α, thereby initiating a “cytokine storm” that induced persistent hyperthermia and resulted in immune- mediated damage to normal tissues. Previous studies have demonstrated that patients with HLH exhibited specific cytokine profiles characterized by significantly elevated levels of IFN-γ and IL-10, along with moderately elevated levels of IL-6 (4). Our findings validated these observations, by demonstrating elevated IL-6 and IL-10 levels in EBV-HLH compared to EBV-IM. IL-6 has been demonstrated to attenuate the cytolytic activity of NK cells. IL-10, expressed by a variety of immune cells, plays a significant role in anti-inflammatory processes. IL-10 disrupts the functionality of antigen-presenting cells (APCs) and downregulates the expression of major histocompatibility complex class II (MHC-II), thereby impairing antigen presentation. Additionally, IL-10 inhibits T-cell proliferation, compromises the secondary responses of CD8+ T cells, and diminishes the production of inflammatory mediators by neutrophils (31). Consequently, it is critical to delineate the cytokine changes in EBV-IM and EBV-HLH following EBV infection, which can aid clinicians in the early identification of EBV-HLH.

Specimens from patients with EBV-HLH exhibit diverse EBV antibody responses, which may indicate either primary EBV infection or reactivation of a previous infection. In this study, anti-EBV-NA-IgG was identified as a significant factor in the predictive model. It is well-established that the host organism sequentially produces antibodies against various EBV antigens (CA, EA, NA) following the infection, with IgM and IgG antibodies against EBV-CA being generated at an early stage, followed by antibodies against EBV-EA. The anti-EBV-NA-IgG typically manifested at a later stage of the infection (32). Consequently, anti-EBV-NA-IgG served as a critical marker for clinicians, not only in pinpointing of the infection timeline, but also in recognition of the progression of EBV-IM into EBV-HLH.

A hallmark of HLH is the hepatic injury-induced release of ferritin, which acts as an acute phase reactant. The diagnostic and prognostic utility of ferritin cut-off values in pediatric HLH has been extensively investigated. The 2004 HLH guidelines, which are widely implemented in routine pediatric practice, include a ferritin level of ≥500 ng/ml as one of the diagnostic criteria for HLH. This threshold was subsequently adjusted to 10,000ng/ml by Allen et al., achieving a sensitivity of 90% (95% CI: 71-100%) and a specificity of 96% (95% CI: 94-98%) (33). In our study, we integrated ferritin with four additional parameters to develop a predictive model for HLH, which proved to be more effective than assessing ferritin levels alone. It is important to note that elevated ferritin is not exclusive to HLH. It is also observed in conditions such as kidney failure, liver disease, infections, and malignancies.

In a study, researchers have found that IL-10, IFN-γ, ferritin and D-dimer levels were significantly different between EBV-HLH and EBV-IM. In the present study, we also found ferritin and IL-10 were independent predictors of EBV-HLH. IFN-γ was subjected to multivariate logistic regression analysis (P=0.809) but was not included in the predictive model. The coagulation related indicators of the most EBV-IM patients were not collected, so D-dimer levels were not adopted in our study (12).

This study has several limitations. First, as a retrospective analysis, we were unable to monitor patients from the onset of fever to the diagnosis of EBV-HLH. Second, due to the single-center nature of this study, the reproducibility of our results necessitates validation through multicenter studies with a larger sample size.

## Conclusion

In summary, IL-6, IL-10, CD3^-^CD16^+^CD56^+^ cells, anti-EBV-NA-IgG and ferritin are potential biomarkers for the early differentiation between EBV-HLH and EBV-IM.

## Data Availability

The raw data supporting the conclusions of this article will be made available by the authors, without undue reservation.
