# Supplementary material for: A predictive model for Epstein-Barr virus-associated hemophagocytic lymphohistiocytosis
Source: Front Immunol. 2024 Dec 5;15:1503118. doi: 10.3389/fimmu.2024.1503118 (PMC11655318; doi:10.3389/fimmu.2024.1503118)
Supplement: Supplementary file 1 [file DataSheet1.pdf]

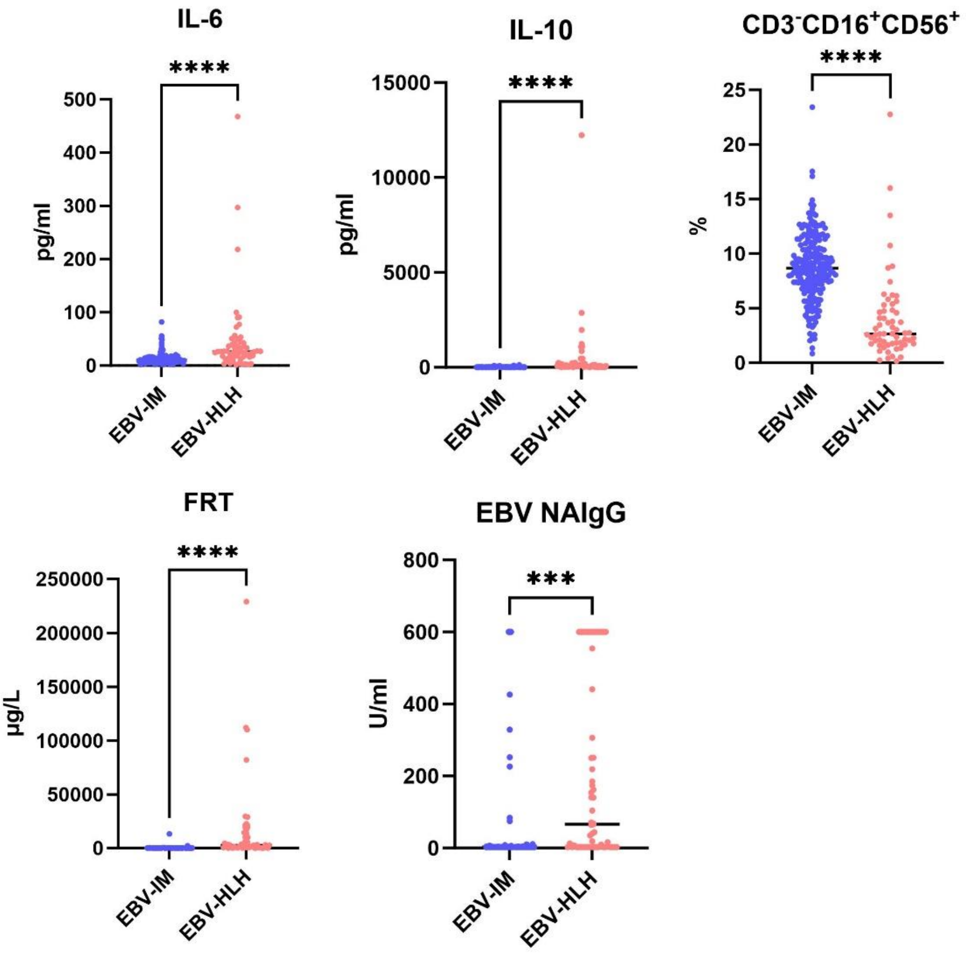

Figure S1 The dotplots showed the different distributions of the five significant blood parameters between EBV-IM and EBV-HLH.
