# Supplementary material for: A predictive model for Epstein-Barr virus-associated hemophagocytic lymphohistiocytosis
Source: Front Immunol. 2024 Dec 5;15:1503118. doi: 10.3389/fimmu.2024.1503118 (PMC11655318; doi:10.3389/fimmu.2024.1503118)
Supplement: Supplementary file 2 [file Table1.docx]

**Table S1 The characteristics of patients in two cohorts.**

| Variables | | Derivation Cohort | Validation Cohort | χ^2^ value | *P*-value |
| --- | --- | --- | --- | --- | --- |
| EBV-HLH | NO | 162 (79.80) | 59 (75.64) | 0.58 | 0.446 |
|  | Yes | 41 (20.20) | 19 (24.36) |  |  |
| Gender | Female | 86 (42.36) | 38 (48.72) | 0.92 | 0.337 |
|  | Male | 117 (57.64) | 40 (51.28) |  |  |
| Age | Age≤2.125 | 35 (17.24) | 9 (11.54) | 1.39 | 0.239 |
|  | Age>2.125 | 168 (82.76) | 69 (88.46) |  |  |
| Ferritin | Ferritin≤250 | 161 (79.31) | 60 (76.92) | 0.191 | 0.662 |
|  | Ferritin>250 | 42 (20.69) | 18 (23.08) |  |  |
| CD3^+^% | CD3^+^%≤91.705 | 190 (93.60) | 73 (93.59) | 0.00 | 1.000 |
|  | CD3^+^%>91.705 | 13 (6.40) | 5 (6.41) |  |  |
| CD3^+^CD4^+^% | CD3^+^CD4^+^%≤21.115 | 141 (69.46) | 55 (70.51) | 0.03 | 0.863 |
|  | CD3^+^CD4^+^%>21.115 | 62 (30.54) | 23 (29.49) |  |  |
| CD3^+^CD8^+^% | CD3^+^CD8^+^%≤54.975 | 62 (30.54) | 22 (28.21) | 0.15 | 0.702 |
|  | CD3^+^CD8^+^%>54.975 | 141 (69.46) | 56 (71.79) |  |  |
| CD4^+^/CD8^+^ | CD4^+^/CD8^+^≤0.315 | 120 (59.11) | 46 (58.97) | 0.00 | 0.983 |
|  | CD4^+^/CD8^+^>0.315 | 83 (40.89) | 32 (41.03) |  |  |
| CD3^-^CD16^+^CD56^+^% | CD3^-^CD16^+^CD56^+^%≤6.295 | 69 (33.99) | 26 (33.33) | 0.01 | 0.917 |
|  | CD3^-^CD16^+^CD56^+^%>6.295 | 134 (66.01) | 52 (66.67) |  |  |
| CD3^-^CD19^+^% | CD3^-^CD19^+^%≤14.02 | 160 (78.82) | 59 (75.64) | 0.33 | 0.565 |
|  | CD3^-^CD19^+^%>14.02 | 43 (21.18) | 19 (24.36) |  |  |
| LDH | LDH≤640 | 167 (82.27) | 65 (83.33) | 0.04 | 0.833 |
|  | LDH>640 | 36 (17.73) | 13 (16.67) |  |  |
| HBDH | HBDH≤458.5 | 167 (82.27) | 65 (83.33) | 0.04 | 0.833 |
|  | HBDH>458.5 | 36 (17.73) | 13 (16.67) |  |  |
| EBV DNA copy No. | EB DNA copy No.<10^3 | 152 (74.88) | 64 (82.05) | 1.63 | 0.202 |
|  | EB DNA copy No.≥10^3 | 51 (25.12) | 14 (17.95) |  |  |
| anti-EBV-VCA-IgM | anti-EBV-VCA-IgM<20 | 12 (5.91) | 4 (5.13) | 0.00 | 1.000 |
|  | anti-EBV-VCA-IgM≥20 | 191 (94.09) | 74 (94.87) |  |  |
| anti-EBV-VCA-IgG | anti-EBV-VCA-IgG<20 | 8 (3.94) | 3 (3.85) | 0.00 | 1.000 |
|  | anti-EBV-VCA-IgG≥20 | 195 (96.06) | 75 (96.15) |  |  |
| anti-EBV-EA-IgG | anti-EBV-EA-IgG<10 | 45 (22.17) | 17 (21.79) | 0.00 | 0.946 |
|  | anti-EBV-EA-IgG≥10 | 158 (77.83) | 61 (78.21) |  |  |
| anti-EBV-NA-IgG | anti-EBV-NA-IgG<5 | 160 (78.82) | 58 (74.36) | 0.64 | 0.422 |
|  | anti-EBV-NA-IgG≥5 | 43 (21.18) | 20 (25.64) |  |  |
| IL-2 | IL-2<2.57 | 168 (82.76) | 61 (78.21) | 0.77 | 0.379 |
|  | IL-2≥2.57 | 35 (17.24) | 17 (21.79) |  |  |
| IL-4 | IL-4<0.775 | 67 (33.00) | 24 (30.77) | 0.13 | 0.720 |
|  | IL-4≥0.775 | 136 (67.00) | 54 (69.23) |  |  |
| IL-6 | IL-6<15.95 | 153 (75.37) | 54 (69.23) | 1.09 | 0.295 |
|  | IL-6≥15.95 | 50 (24.63) | 24 (30.77) |  |  |
| IL-10 | IL-10<26.515 | 155 (76.35) | 59 (75.64) | 0.02 | 0.900 |
|  | IL-10≥26.515 | 48 (23.65) | 19 (24.36) |  |  |
| TNF-α | TNF-α<1.210 | 57 (28.08) | 26 (33.33) | 0.75 | 0.387 |
|  | TNF-α≥1.210 | 146 (71.92) | 52 (66.67) |  |  |
| IFN-γ | IFN-γ<12.87 | 158 (77.83) | 60 (76.92) | 0.03 | 0.870 |
|  | IFN-γ≥12.87 | 45 (22.17) | 18 (23.08) |  |  |
